# Effect of Surface Impurities and Lattice Defects on the Photocatalytic Activity of ZnO Nanoparticles

**DOI:** 10.1021/acs.langmuir.5c03385

**Published:** 2025-10-16

**Authors:** Fredric G. Svensson, Erik Djurberg, Seohan Kim, Gunnar Westin, Lars Österlund

**Affiliations:** 1 Department of Materials Science and Engineering, The Ångström Laboratory, 8097 Uppsala University, Box 35, Uppsala 751 03, Sweden; 2 Department of Materials Science and Engineering, Pusan National University, Busan 46 241, South Korea; 3 Department of Chemistry, The Ångström Laboratory, 8097 Uppsala University, Uppsala 751 21, Sweden; 4 Department of Chemistry, Umeå University, Umeå 901 87, Sweden

## Abstract

We report on the surface chemical and photocatalytic properties of hierarchical ZnO nanorod assemblies synthesized via zinc acetate-based methods and subjected to various pretreatment protocols. The as-prepared ZnO samples were thoroughly washed and thermally treated at different temperatures and durations to remove residual organics from the synthesis. Despite extensive washing, diffuse reflectance infrared Fourier transform spectroscopy (DRIFTS) and X-ray photoelectron spectroscopy revealed significant amounts of residual organic species on the as-prepared ZnO surfaces. Operando DRIFTS demonstrated that annealing to 500 °C was required to effectively eliminate these synthesis-related residues. However, prolonged annealing also led to the removal of two types of ZnO-associated defects: (i) terminal −OH groups, which reformed upon cooling in synthetic air, and (ii) intrinsic lattice defects hosting bridge-bonded OH species, which were irreversibly healed. The catalytic performance of the ZnO samples, assessed by phenol photodegradation and its decomposition products, showed enhanced activity for washed and calcined ZnO (up to 52% degradation) compared to as-prepared samples treated only by chemical washing (24% degradation). Operando DRIFTS revealed that extended thermal treatment at 500 °C led to a decline in catalytic activity (34% degradation) relative to samples exposed to shorter annealing times. This loss in activity was directly correlated with a decreased concentration of catalytically active lattice vacancy defects. Our findings provide molecular-level evidence of two competing effects during thermal pretreatment of ZnO in oxidizing environments: the removal of inhibitory organic residues (which enhances activity) and the healing of reactive lattice defects (which reduces activity). These results underscore the critical importance of optimizing pretreatment conditions when designing efficient ZnO-based catalysts from organic zinc precursors and highlight the essential role of lattice defects in governing ZnO surface reactivity.

## Graphical abstract

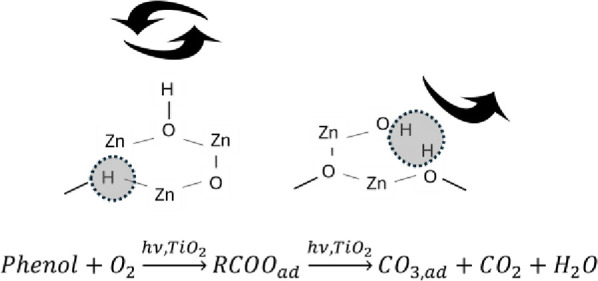

## Introduction

Zinc oxide (ZnO) is extensively used in a wide range of products and technical applications including filler materials, light emitting diodes, sensors, and as a white pigment in paints.^1,2^ Its strong absorption of ultraviolet (UV) light has led to its use as a UV-blocker in, e.g., sunscreen lotions.^3^ It is a widely used catalyst in a variety of reactions, notably as a hydrogenation catalyst for methanol synthesis (with Cu and alumina, CZA),^4^ which can be related to its chemical properties, actively participating in the surface chemistry of the CZA catalysts by pronounced metal–support interactions. ZnO is also the most studied photocatalyst material after TiO_2_.^5–7^ ZnO is a direct bandgap wide-bandgap semiconductor with an approximate bandgap of 3.4 eV, depending on size and morphology, with high exciton binding energy (about 60 meV). ZnO exists in the wurtzite structure at most conditions being studied. Its electronic properties along with the relative simpleness to synthesize wurtzite ZnO have thus led to many applications in electronics and optoelectronics. There is extensive literature on the electronic properties of ZnO.^8–10^

There are many routes to prepare ZnO nanoparticles from molecular Zn precursors, for instance reflux in organic media,^11^ thermal decomposition,^12^ sol–gel,^13,14^ and hydro/solvothermal.^15^ The most common precursors are acetate, nitrate, and chloride salts of zinc. Under ambient or near-ambient solution-based synthetic conditions, the hexagonal wurtzite crystal phase is the most energetically favorable form where each Zn^2^^+^ cation is tetrahedrally coordinated by four O^2^^–^ anions, and vice versa.^8^ Because of the highly anisotropic structure of the hexagonal wurtzite phase with a preferred growth along the *c*-axis, ZnO easily grows in rod- or plate-like structures that can form elaborate hierarchical 3D assemblies, e.g. “flowers”, “sea urchins”, etc. These hierarchical 3D structures have attracted much attention for use in various technological applications, including sensors and (photo)­catalysis.^6,15^ Efforts have been made to develop and further control the structures by controlling the synthetic conditions. It is now known that factors like the Zn^2+^:OH^–^ ratio (pH), organic additives, and solvent mixtures (e.g., H_2_O:EtOH) have critical effects on the formation and self-assembly of ZnO.^1,16–18^ The total area of high-energy crystal facets is minimized during particle growth if sufficient energy is provided in the reaction. For wurtzite ZnO, these surfaces are the polar Zn-terminated (0001) surface, which is positively charged, and the O-terminated (0001̅) surface, which is negatively charged. By the addition of ligands that are selective for specific facets, it is possible to change the relative energies of the ZnO faces and promote growth of high-energy surfaces.^19^

In many applications such as heterogeneous (photo)­catalysis and solid-state sensors, the surface chemistry of ZnO is crucial. Adsorbed surface species originating from the synthesis chemicals can bond to the surface atoms, which eventually may poison the catalytic effect and block adsorption of the analyte to be detected by the sensor. Many small organic molecules, including water, are known to readily adsorb, dissociate, and transform into various (strongly bonded) products on and inside the ZnO structure.^20^ On an atomistic scale, the observed activity of a catalyst depends on so-called active sites. These do not necessarily correspond to the number of available surface atoms of the catalyst. Often low-coordinated surface atoms and missing lattice atoms, generally called defect sites, play an important role, which can be minority sites only occupying a small fraction of the exposed catalyst surface. Inhibition of active sites by reaction products, or by catalyst synthesis residues, can block active sites. This is particularly important in low-temperature catalysis, where photocatalysis is a typical example. ZnO typically exhibits native (intrinsic) oxygen vacancy (V_O_) and interstitial Zn (Zn_i_) defects that typical employ some heat treatments.^9,21–23^ Reductive treatment by, e.g., H_2_, which is relevant for methanol synthesis catalysis, results in defects associated with deep-level bandgap states that react with hydrogen and form Zn–H species.^9,24,25^ Defects have been reported to enhance the photocatalytic activity of ZnO,^26–28^ although atomistic reasons that rationalize these findings are still missing.

Because water is always present in air and is formed by chemical reactions on metal oxide surfaces, their interactions with metal oxide surfaces and defects contained in them have received considerable attention. Hydroxyls are known to have pronounced effects on the chemical activity and electronic properties of oxide surfaces.^26–31^ In the case of ZnO, molecular adsorption of water at room temperature leads both to molecular and dissociative adsorption surfaces but also to dissociation of H_2_O forming several types of hydroxyl species.^23,29,32^ Further, nanostructured ZnO exposes low-coordinated surface sites and defects that react with water, hydrogen, and its dissociation products leading to Zn–O and Zn–OH species with different coordination to the surface and its defects.^24,25,33^

We have here undertaken a comprehensive study to investigate the surface chemistry of ZnO prepared by a low-temperature route using a commonly employed metal organic salt, namely zinc acetate, as a precursor. We have studied the thermal treatment under oxidizing conditions of as-prepared ZnO (washed by deionized water only) and measured the concentration and type of surface residue by means of operando DRIFTS as a function of temperature. In addition, we have measured the photocatalytic activity of the as-prepared and annealed ZnO samples. We have chosen to study sea urchin-like ZnO hierarchical structures since they form large particles rich in intrinsic surface defects. Based on our findings, we conclude that ZnO nanoparticles prepared by metal organic precursors, such as zinc acetate, contain significant amounts of defects and organic synthesis residues that, if not properly removed, significantly affect their catalytic activity.

## Materials and Methods

### Synthesis of Sea Urchin-like ZnO Nanoparticles

ZnO nanorods were synthesized by a simple low-temperature method. Briefly, 0.27 M zinc acetate dihydrate (reagent grade, Sigma-Aldrich) solution in deionized (DI) water was added to a 2.73 M sodium hydroxide solution in deionized water. The Zn^2+^:OH^–^ molar ratio was 1:10. Upon mixture, an initial white precipitate of Zn­(OH)_2_ (s) forms that quickly dissolves upon mixing to yield a clear, colorless solution of [Zn­(OH)_4_]^2–^ (aq). The solution was placed in a water bath preheated to 50 °C and kept for 90 min. After about 10 min, the solution turned slightly opaque, and after 90 min, a white precipitate of ZnO was formed. The precipitate was separated by decantation and subsequently washed with DI water and centrifuged repeatedly until neutral pH of the supernatant was reached (about seven cycles). Guided by the results from our pretreatment preparation experiments, some of the ZnO was purposefully heated at 500 °C for 10, 30, or 60 min, employing a 20 min ramp and natural cool-down to remove surface-bound organics and improve crystallization to achieve well-developed ZnO facets.

### Characterization of Materials

#### X-ray Diffraction

Powder X-ray diffraction (PXRD) was used to determine the crystal phase and particle size of the powders. A Siemens D5000 powder X-ray diffractometer with Cu Kα_I,II_ radiation (λ = 1.5418 Å) in Bragg–Brentano geometry was used. A step size of 0.02° and divergence- and antiscattering slit widths of 1° were used. From the diffractograms, the average crystallite sizes were estimated by the Scherrer equation:

$$D=\frac{K\lambda }{B_{c}\;\cos\;\theta }$$

where *D* is the average crystallite size, *K* is a shape factor (here 0.94, appropriate for rod-shaped particles), *B*_c_ is the full width at half-maximum (fwhm) corrected for instrumental broadening (NIST 1976 Al_2_O_3_ standard), and θ is the Bragg angle in radians. The crystallite sizes for ZnO were calculated as an average of the fwhm of the (100), (002), (101), (102), (110), (103), (200), (112), and (201) planes. The fwhm values were obtained from peak fitting of the diffractograms using the software Origin 2019.

#### Electron Microscopy

Morphological and structure analysis of the ZnO samples was performed with a Zeiss LEO 1550 scanning electron microscope (SEM) equipped with a field-emission gun operated at 3–8 keV. All powder samples were dispersed on carbon tape for analysis. Transmission electron microscopy (TEM) was performed using a field-emission transmission electron microscope (FE-TEM, JEOL model JEM-2100F). Selected-area electron diffraction (SAED) was performed in selected areas identified by high-resolution TEM. Lattice spacings were quantified by performing phase identification using ZnO references in the PDF-5+ database, yielding ICDD 04-06-1673 as the best fit to the experimental data. The experimental data were then calibrated by using the unit cell dimensions of the database reference. Fourier transform filtered bright-field high-resolution images were produced from the calibrated diffraction data.

#### Diffuse Reflectance Infrared Fourier Transform Spectrometry

In situ diffuse reflectance infrared Fourier transform spectrometry (DRIFTS) was used to investigate the presence of vibrational bands due to residual organic ligands and defects of the as-prepared and heat-treated ZnO as a function of temperature and time employing a ramp rate of 25 °C min^–1^. A Vertex 80 FTIR spectrophotometer (Bruker Optics, Ettlingen, Germany), equipped with a custom modified HPHT reaction cell placed in a Praying Mantis DRIFTS accessory (Harrick Scientific Products, Inc., NY, USA, was used for these experiments,^34^ employing a liquid nitrogen-cooled HgCdTe detector. About 30 mg of pure, unmixed ZnO powder was used in each experiment. Background spectra were collected on each ZnO powder at room temperature in synthetic air after about 15 min. Spectra were acquired from 16 scans using 4 cm^–1^ spectral resolution. All spectra were smoothed with a Savitzky–Golay algorithm using a 7-point window. Deconvolution of FTIR peaks was performed by fitting of the experimental curves with Gaussian functions.

#### Raman Spectroscopy

Raman spectroscopy was performed with a Horiba LabRam HR800 employing a 532 nm excitation laser. An 1800 grooves mm^–1^ grating and a thermoelectric air-cooled CCD detector were used in all measurements. A 10× objective (Olympus MPlan N objective) was used to focus the laser light onto the sample and to collect the 180° backscattered light. Raman spectra were acquired for 30 × 10 s. ZnO powder samples were placed on microscope glass slides covered in aluminum foil.

#### X-ray Photoelectron Spectroscopy

A Quantera II scanning electron microscope from Physical Electronics utilizing an Al Kα X-ray source was employed to characterize the surfaces of the different ZnO samples. A low-energy flood gun, operating at 1.0 V and 20.0 μA, was employed for charge compensation. For the survey spectra, a pass energy of 224 eV and a resolution of 0.8 eV were used, and for the high-resolution spectra, a pass energy of 55 eV and 0.1 eV resolution were employed. Sample powders were prepared on a glass slide by suspending a water suspension that was dried under a desktop lamp. The binding energy was calibrated against the C 1s peak at 284.8 eV from adventitious carbon and carefully checked for possible charging effects by varying the sample presentation and flood gun energy. Data were treated and analyzed using CasaXPS software.^35^ The spectra were smoothed by applying a Savitzky–Golay algorithm using a 5-point window.

#### Surface Area

The specific surface areas of the four samples were determined using a Micromeritics ASAP 2020 instrument employing BET analysis. Nitrogen physisorption measurements were performed on approximately 100 mg of samples using a liquid nitrogen bath. The samples were predried in a furnace at 120 °C for 2 h and then stored in sealed plastic vessels in a desiccator before degassing under vacuum at 120 °C for 2 h in a Micrometrics SmartVacPrep system prior to analysis.

#### Photodegradation of Phenol

The photocatalytic activities of the materials were evaluated for the degradation of phenol (Sigma-Aldrich, 99+%) under UV illumination using a UV diode laser source (λ = 365 nm, fwhm = 12 nm; 19 mW/cm^2^, Prizmatix Ltd., Holon, Israel) placed 1 cm over the cuvette. 1.5 mg of catalyst powder was added to 3 mL (equal to 0.5 g/L catalyst) of 0.64 mmol/L phenol solution in a standard quartz cuvette (1 cm side length, 4 mL capacity). A 5 mm Teflon-coated magnetic bar was used to stir the suspension at 300 rpm, which resulted in a slightly opaque solution, but clear enough for in situ UV spectrophotometric measurements. After mixing the catalyst powder with the phenol solution, the suspension was stored in darkness for 30 min prior to the photocatalytic test started. In situ absorption spectra were collected using an HR2000+ spectrophotometer (Ocean Optics) with a deuterium and halogen light source (Micropack). The spectral range between 190 and 1100 nm was measured using an average of 100 consecutive scans with a measurement time of 2 ms. The area of the integrated absorption band for phenol occurring at about 271 nm was used to follow the degradation. UV absorbance due to ZnO did not interfere with the phenol absorbance band (Figure S1), which is consistent with a previous report that the absorption onset for larger colloidal ZnO nanoparticles in aqueous solutions, as in our case, occurs above about 370 nm.^36^ The UV LED emits very little heat, and no temperature increase in solvent evaporation was noticed in the cuvette during catalytic experiments. This assertion can be quantified as follows. If we assume only natural convective losses and ignore evaporate cooling and radiative losses, then the photon power is balanced by convection as follows: *P* = α*lhA*Δ*T*, where *P* = 0. 01 W is the photon power, α ≈ 0.02 cm^–1^ is the absorption coefficient of water at 365 nm, *l* = 1 cm is the path length in the cuvette, *h* ≈ 5 W/m^2^ K is the convection coefficient, *A* = 1 cm^2^ is the illuminated area, and Δ*T* is the steady-state temperature rise. This yields a negligible estimated temperature rise Δ*T* ≈ 0.4 K.

The phenol absorption band centered at 271 nm was integrated using Origin 2019 software with a user-defined baseline. UV spectrophotometric measurements were done at times −30, 0, 15, 30, 45, 60, 90, 120, 150, and 180 min, defined relative to the starting point of the UV illumination. The catalytic experiments were performed in triplicates for all four ZnO catalyst samples (as-prepared sample and samples annealed at 500 °C for 10, 30, and 60 min). Degradation plots are presented as normalized *C*/*C*_0_ versus time, where *C*_0_ is the integrated absorbance at 0 min. The absolute degradation rates were calculated from the amount of degraded phenol (defined by the decrease in the phenol absorption band at 271 nm) per unit surface area of the ZnO catalyst (determined from the measured specific surface area).

## Results and Discussion

ZnO nanoparticles were obtained from a low-temperature synthesis route employing zinc acetate dihydrate without any additional organic additives. The as-prepared, white powders were crystalline in the wurtzite phase (hexagonal space group *P*63*mc*) (Figure 1). Analysis of XRD data in Figure 1 shows that heat treatment at 500 °C for 10, 30, and 60 min in air improved the crystallinity and increased the crystallite size. However, the crystallite growth slowed down after 10 min and only grew marginally up to 60 min. Average crystallite sizes and associated standard deviation for the as-prepared, 10, 30, and 60 min annealed samples were calculated to be 36.9 ± 12, 45.8 ± 14, 46.0 ± 14, and 47.4 ± 15 nm, respectively, using the Scherrer equation (Table 1). No apparent increase in average particle size was observed when drying the as-prepared ZnO at 120 °C prior to the surface area measurement. The ratio of the integrated intensity of (002) and (100) reflections, where (002) corresponds to the polar surface and (100) the nonpolar surface, gives an estimate of the amount of polar high-energy facets. This ratio is important since it can affect the photocatalytic activity. A higher *I*_(002)_/*I*_(100)_ ratio has been reported to correlate with higher photocatalytic activity.^37^ The *I*_(002)_/*I*_(100)_ ratios for ZnO as-prepared, ZnO 10 min, ZnO 30 min, and ZnO 60 min were calculated to be 0.683, 0.722, 0.714, and 0.710, respectively, implying a slightly higher amount of polar high-energy facets (about 5%) for the annealed ZnO samples compared with the as-prepared sample.

**Table 1:** Summary of the Physical and Catalytic Properties of the Four Different ZnO Samples

ZnO sample	crystal phase	crystallite size (nm)	XRD intensity ratio *I*_(002)_/*I*_(100)_	BET surface area (m^2^/g)	Raman intensity ratio (*E*_1Lo_)/(*E*_2High_)	XPS intensity ratio *I*_(530.4)_/*I*_(529.0)_	integrated IR absorbance (cm^–1^)
as-prepared	wurtzite	35.3 ± 11	0.683	7.1 ± 0.2	0.095	0.52	2.73
10 min at 500 °C	wurtzite	43.9 ± 13	0.722	6.3 ± 0.5	0.050	0.57	
30 min at 500 °C	wurtzite	44.0 ± 13	0.714	6.1 ± 0.2	0.048	0.50	0.73
60 min at 500 °C	wurtzite	45.4 ± 14	0.710	8.6 ± 0.4	0.00015	0.42	

**Figure 1:**
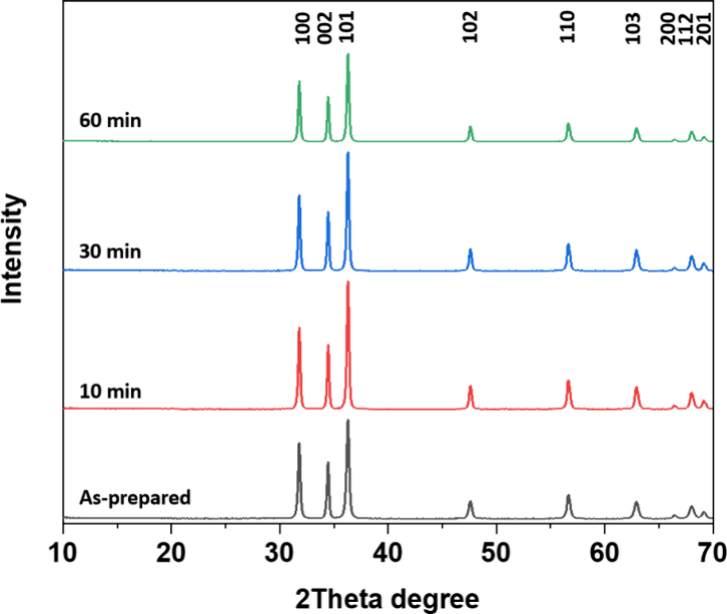
Indexed ZnO diffraction patterns for as-prepared, 10, 30, and 60 min heat-treated samples at 500 °C. All ZnO patterns correspond to the reference pattern for the wurtzite phase (ICCD no. 04-006-1673).

The specific surface areas were determined to be approximately 7, 6, 6, and 9 m^2^/g for as-prepared ZnO and ZnO heated for 10, 30, and 60 min, respectively, (Table 1), suggesting no major changes of exposed geometrical samples areas. These values agree with other reports of ZnO with similar structures and preparation methods.^17,18^ We note, however, that these surface areas are in the lower end of what can typically be measured reliably by the BET method.

The morphology of the as-prepared and heat-treated ZnO powder samples was investigated by electron microscopy. Figure 2a,b shows representative SEM micrographs of as-prepared and ZnO samples heat-treated at 500 °C for 30 min. The low-temperature method yielded sea urchin-like assemblies consisting of needle-like crystalline ZnO nanoparticles, which are clearly seen in the TEM images. The ZnO sea urchins had an average diameter of about 5 μm. No apparent morphological changes or aggregate growth occurred during the heat treatment according to the SEM micrographs. High-resolution TEM analyses indicate that both the as-prepared and annealed ZnO nanoparticles are crystalline. It is evident that more developed crystalline structures exhibiting homogeneous morphology and distinct diffraction patterns are observed after heat treatment (Figure 2c,d). No residual sodium ions could be detected neither from XPS nor EDS analysis; however, there are still substantial amounts of carbonates present, particularly for the as-prepared ZnO. The presence of sodium could result in the formation of sodium carbonate, with a subsequent negative impact on photocatalytic activity.

**Figure 2:**
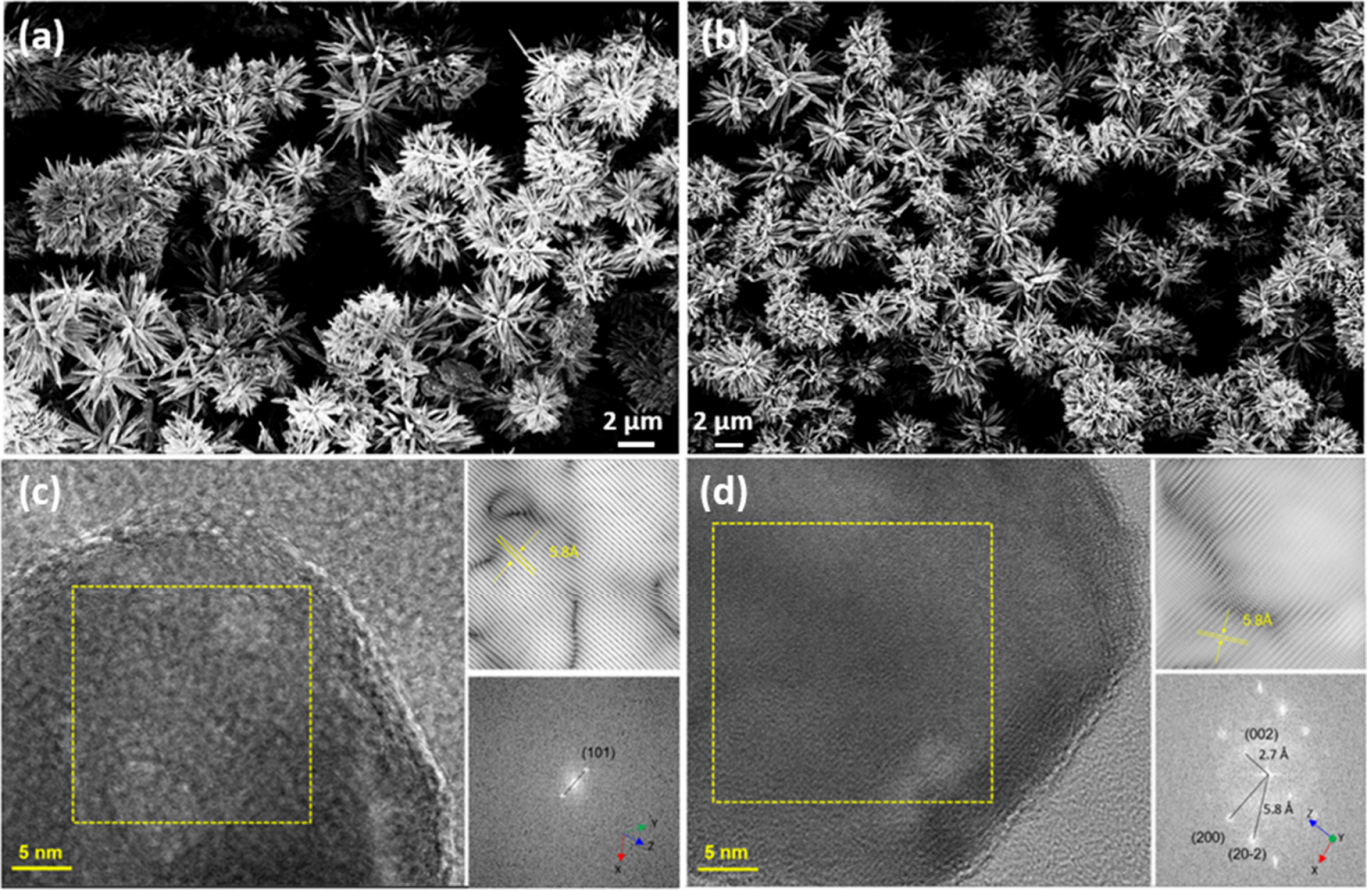
Representative SEM micrographs of (a) as-prepared ZnO “sea urchins” and (b) ZnO “sea urchins” heat-treated at 500 °C for 30 min. High-resolution TEM micrographs of the rod-like part of (c) as-prepared ZnO and (d) ZnO annealed at 500 °C for 30 min, with the lattice fringe distance for the (101) direction indicated.

Raman spectra were recorded for as-prepared ZnO and heat-treated ZnO (500 °C for 30 min). The spectra for as-prepared and heat-treated ZnO are shown in Figure 3. The observed Raman modes are assigned according to Thyr et al.^38^ It is evident that crystallinity improved after the heat treatment yielding pronounced *E*_2_(low) (at 94–99 cm^–1^) and *E*_2_(high) (at 433–438 cm^–1^) modes due to the alternate or concerted, respectively, “out-of-chain” vibrations of the Zn and O atoms in the −Zn–O–Zn–O– chains running in the (0001) direction in the wurtzite structure, indicating improved crystallization of the rod-like ZnO structures that are oriented in the (0001) direction. The presence of A_1_ modes confirms the presence of the polar surfaces in agreement with the TEM results in Figure 2c,d. The *E*_1Lo_ mode is observed at ∼660 cm^–1^. The *E*_1Lo_ mode has previously been associated with the presence of oxygen vacancies, and the ratio (*E*_1Lo_)/(*E*_2High_) has been used to estimate the amount of oxygen vacancy distribution relative to the crystallinity, although we infer that in several studies, the A_1_ mode was used in the analysis.^39^ The intensity ratio (*E*_1Lo_)/(*E*_2High_) was calculated from the integrated area of the two bands to 0.095, 0.050, 0.048, and 0.00015 cm^–1^ for as-prepared ZnO and ZnO heat-treated for 10, 30, and 60 min, respectively, suggesting no detectable changes of the (*E*_1Lo_)/(*E*_2High_) intensity ratio as a function of heating time within the experimental uncertainty.

**Figure 3:**
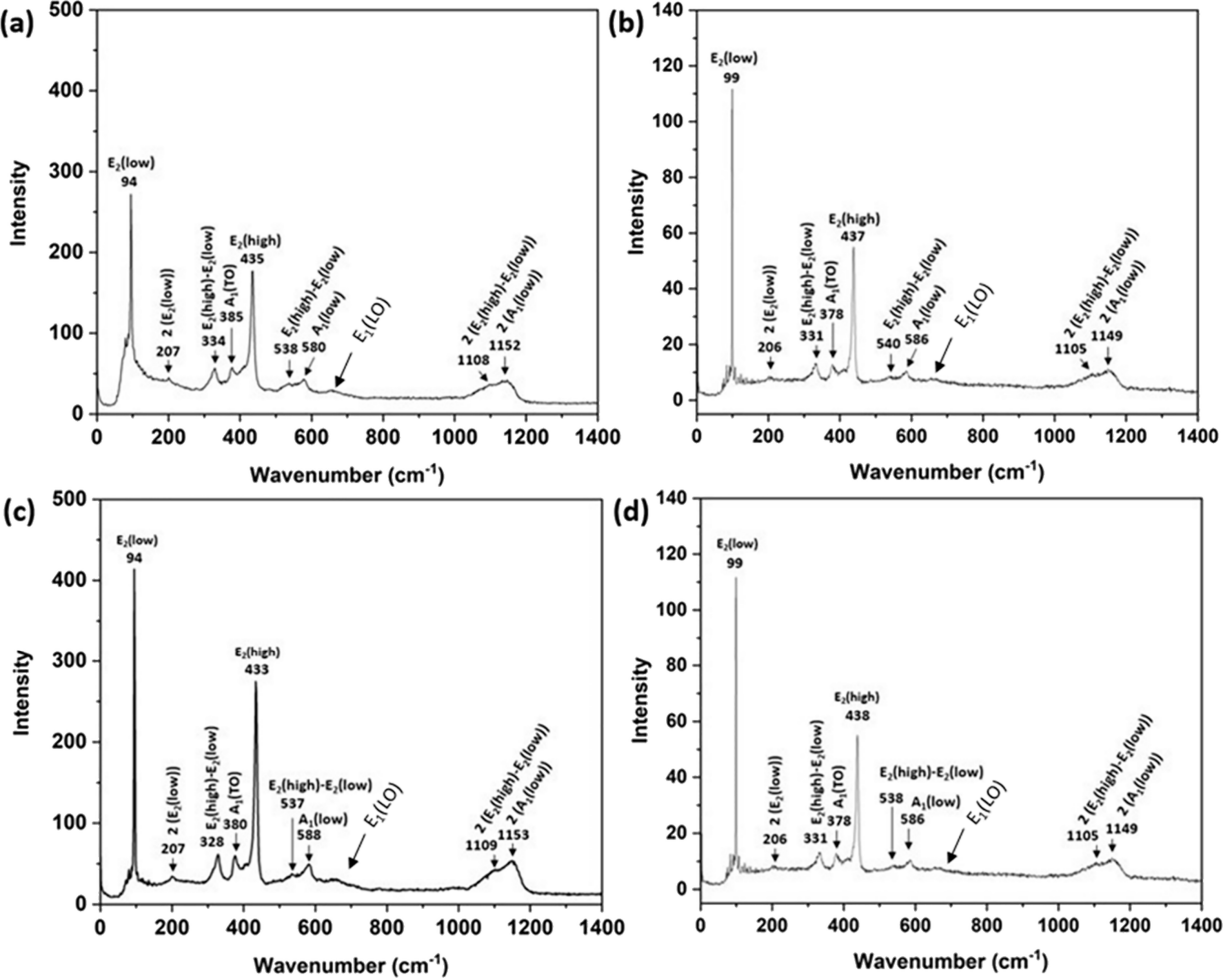
Raman spectra of (a) as-prepared ZnO and (c) ZnO annealed at 500 °C for 10 min, (c) ZnO heat-treated at 500 °C for 30 min, and (d) ZnO heat-treated at 500 °C for 60 min with the different vibration modes indicated.

The ZnO samples were analyzed by high-resolution XPS to get a better understanding of the surface properties of the different treatments. Figure 4 shows high-resolution XPS spectra of the Zn 2p_1/2_ and 2p_3/2_ peaks located at about 1021.2 and 1044.3 eV, yielding a spin–orbit split of 23.1 eV in good agreement with literature data.^40^ A slightly lower binding energy of the Zn 2p peaks for as-prepared ZnO can, however, be noticed compared with the heat-treated samples. Similar Zn 2p binding energies have been reported for sea urchin-like ZnO in other works and may be structure-related.^41,42^ A downshift of the Zn 2p core level is expected for reduced Zn, i.e., surface Zn and oxygen vacancies. Both are likely to be present at higher concentrations on the high-surface-area ZnO sea urchin structures than reference ZnO samples.

**Figure 4:**
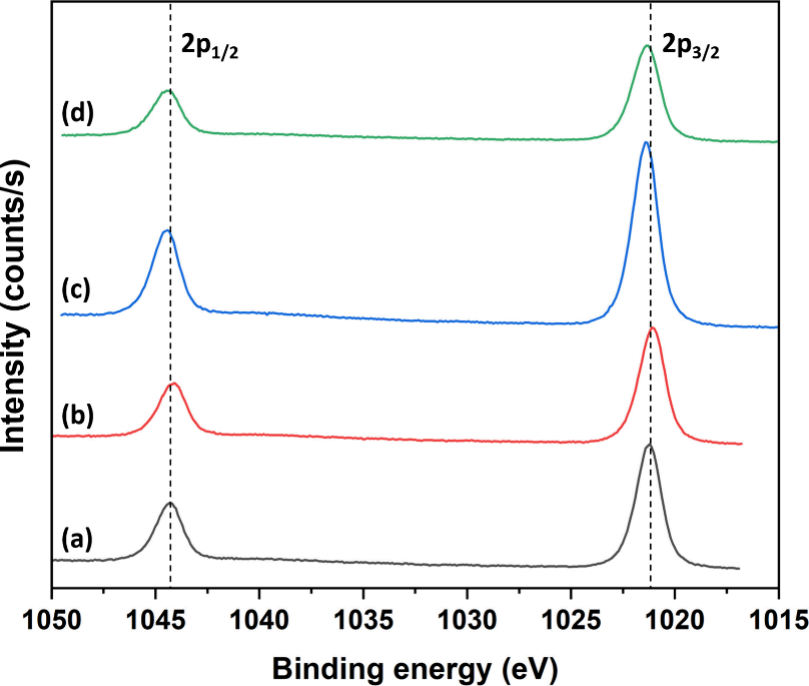
High-resolution Zn 2p XPS spectra of (a) as-prepared ZnO, (b) ZnO annealed at 500 °C for 10 min, (c) ZnO annealed at 500 °C for 30 min, and (d) ZnO annealed at 500 °C for 60 min. The 2p_1/2_ and 2p_3/2_ distance is about 23 eV, in agreement with the literature.^40^

The heat treatment heals oxygen vacancies and improves crystallinity and removes OH groups, which all are expected to shift the binding energy upward, which is also seen in our spectra (an upshift of up to about 0.2 eV after heat treatment of the as-prepared ZnO). The O 1s shift can be deconvoluted into two components, a lower energy binding peak (*L*) and a higher energy binding peak (*H*), positioned at about 530 and 531 eV, respectively, for the different ZnO samples (Figure 5). The higher energy shift, ∼531 eV, is indicative of oxygen vacancies while the lower is attributed to lattice oxygen.^39^ Taking the area ratios of *H*/*L* for as-prepared ZnO and ZnO annealed for 10, 30, and 60 min gives 0.52, 0.57, 0.50, and 0.42, respectively, suggesting a decrease in surface defects after 60 min of heat treatment, which is also consistent with a concomitant Zn 2p binding energy upshift seen in Figure 4 (cf. Figure 4a and d). Referring to the Raman data in the preceding section, we tentatively attribute the downshift in the as-prepared ZnO to oxygen vacancies.

**Figure 5:**
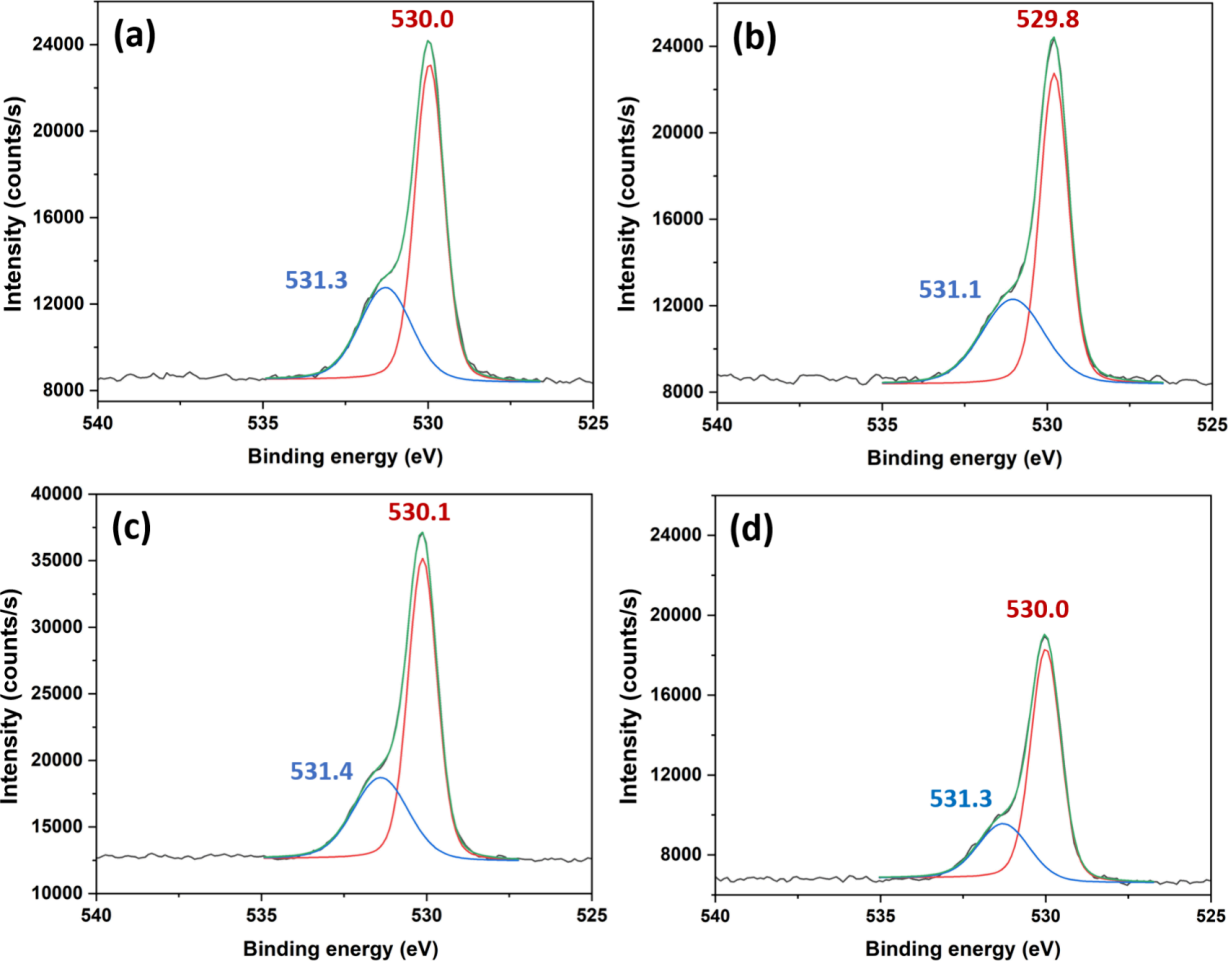
High-resolution O 1s XPS spectra of (a) as-prepared ZnO, (b) ZnO annealed at 500 °C for 10 min, (c) ZnO annealed at 500 °C for 30 min, and (d) ZnO annealed at 500 °C for 60 min.

Since zinc acetate was used as a precursor, it is reasonable to assume that the as-prepared ZnO contains surface-coordinated acetate species and decomposition products thereof, e.g., carboxylates and carbonates. Such surface-bonded synthesis residues can block catalytic sites at the ZnO surface and engage in chemical reactions with reactant molecules in catalytic reactions. Moreover, these residues can also trap electron–hole pairs in photocatalytic reactions. Thus, the amounts of residual organics on as-prepared ZnO and ZnO heat-treated at 500 °C at different periods of time (10, 30, and 60 min) were investigated by in situ DRIFTS while heating the samples to 450 °C and monitoring the formation/disappearance of IR bands characteristic of organic compounds.

Figure 6 shows an overview of stacked in situ DRIFT spectra as a function of temperature for as-prepared and postheated ZnO. From the IR spectra in Figure 6, it is evident that the as-prepared ZnO contains substantial amounts of organic residues despite repeated washing cycles with deionized water. As expected, the loss of OH/H_2_O is much larger from the as-prepared sample compared to the annealed sample (Figure 6a,c), which was also inferred from XPS. On the other hand, comparing the as-prepared ZnO sample to the sample postheated to 500 °C shows that substantial organic surface residues are removed from the as-prepared ZnO during the heating ramp (compare the first and last spectra acquired at 20 °C before and after the in situ heating cycle). This is not the case for the postheated ZnO sample. Heat treatment at 500 °C for 30 min was shown to be sufficient to remove most surface organics, while not leading to major changes of the physical properties of the sample (see Table 1).

**Figure 6:**
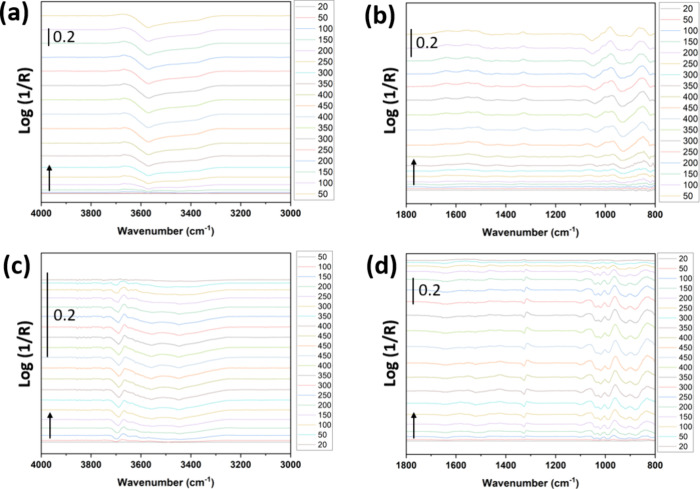
Stacked in situ DRIFT spectra of (a, b) as-prepared ZnO and (c, d) postheated ZnO at 500 °C for 30 min as obtained at different temperatures between 20 and 450 °C during the heating ramp at 25 °C min^–1^. Spectra (a) and (c) show the O–H stretching region due to OH/H_2_O. Spectra (b) and (d) show the fingerprint region of 800–2000 cm^–1^ showing absorbance from primarily C–O bond modes due to organic surface species. The black arrows in the panels indicate the direction of the sequence of time-series DRIFT spectra.

Figure 7 shows detailed DRIFT spectra corresponding to Figure 6 in the fingerprint region along with mode assignments of vibrational bands based on a previous report.^24^ The bands at 1500–1600 and 1350 cm^–1^ can be assigned to the asymmetric and symmetric, respectively, carboxylate bands (−O=C–O−) for acetate and formate,^43^ which is expected as a result of the zinc acetate precursor synthesis route (acetate and its decomposition product, formate). A transformation from acetate to formate can be discerned upon heat treatment where the band closer to 1600 cm^–1^ decreases with the band closer to 1500 cm^–1^. The band around 1040 cm^–1^ is due to corresponding C–O vibrations. Weak bands in Figure 7 at about 1450 cm^–1^ are due to carbonate bands formed through the oxidation of acetate via formate, which is typical for metal oxide surfaces.^43–45^

**Figure 7:**
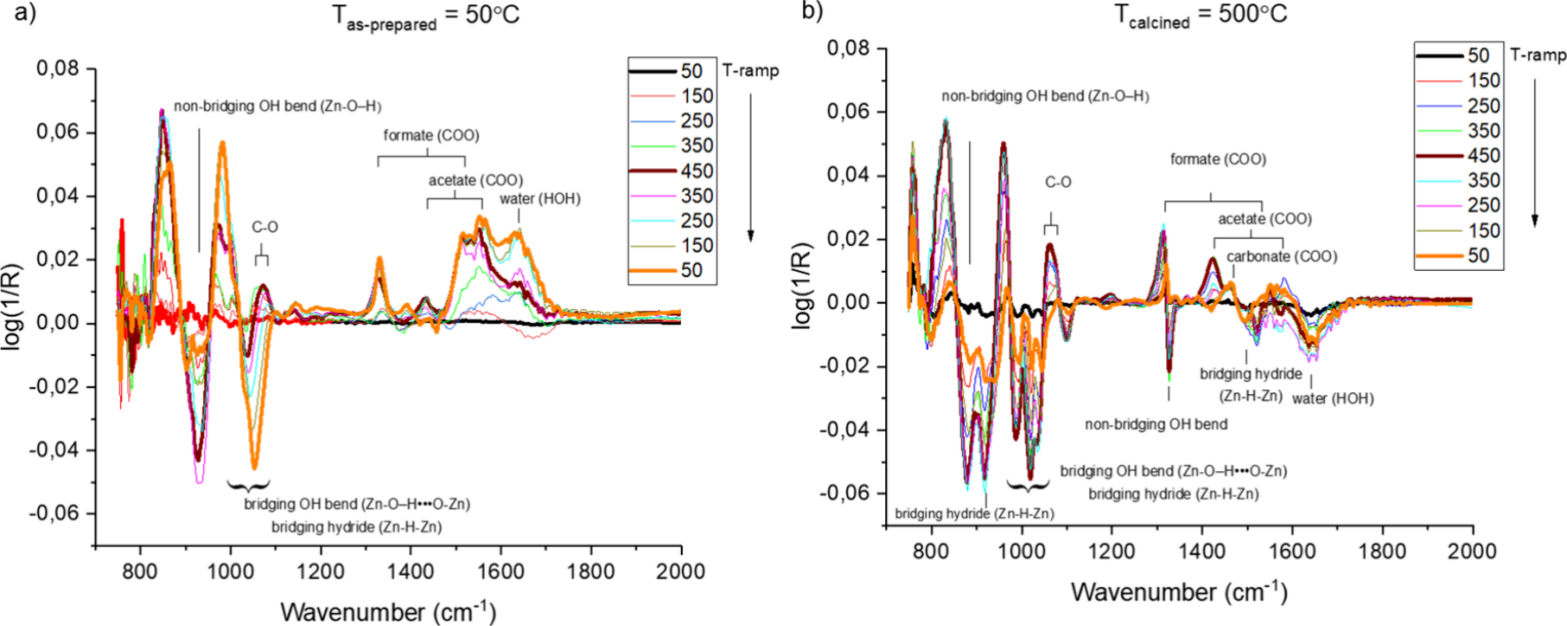
DRIFT spectra in the fingerprint region of (a) as-prepared ZnO and (b) ZnO annealed at 500 °C for 30 min with mode assignments obtained at different temperatures during heating the samples in synthetic air employing a heating ramp of 25 °C min^–1^.

Figure 8 shows the integrated carboxylate bands due to acetate and formate in Figure 7 as a function of temperature. Figure 8 thus shows the remaining concentration of carboxylate species after heat treatment to 450 °C (the limit of our reaction cell setup for extended heat treatment experiments). The amount of removed carboxylate for the as-prepared ZnO was calculated to be 2.73 cm^–1^, while for the ZnO annealed at 500 °C for 30 min, it was calculated to be 0.73 cm^–1^, showing that the thermally pretreated sample contains much less surface carboxylates. The remaining fraction of carboxylates for the postheated sample in Figure 7b can largely be attributed to incomplete oxidation at 450 °C of residual hydrocarbons that are adsorbed on all ZnO powders after preparation and storage in air since this fraction remains the same also after >30 min of heat treatment at 500 °C. On the other hand, substantial carboxylate builds up on the as-prepared sample, which can be attributed to gradual oxidation of precursor acetate synthesis residues.

**Figure 8:**
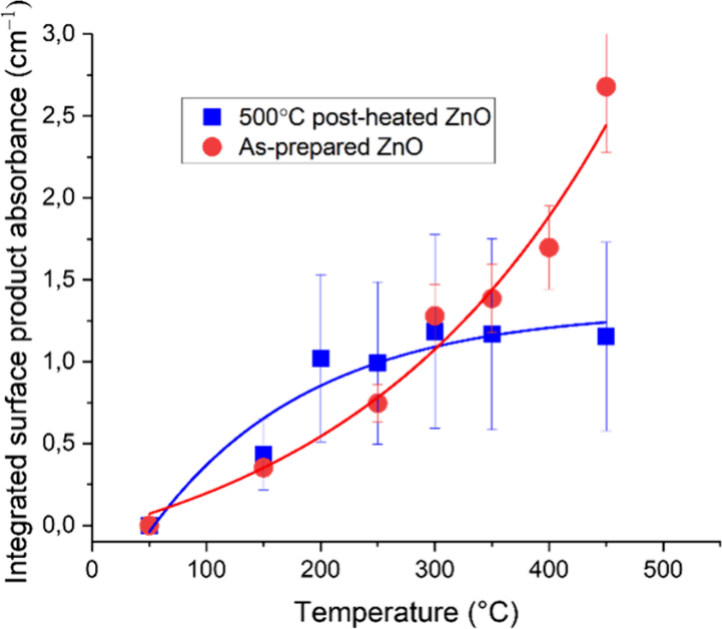
DRIFT spectra in the fingerprint region of (a) as-prepared ZnO and (b) ZnO annealed at 500 °C for 30 min with mode assignments obtained at different temperatures during heating the samples in synthetic air employing a heating ramp of 25 °C min^–1^.

The IR bands in the 850–950 cm^–1^ region in Figure 7 due to O–H bands due to nonbridging OH bending modes and bridging hydrides associated with lattice defects can be seen.^24^ Notably, the bent bridging OH groups around 1025 cm^–1^ disappear on the as-prepared ZnO as a function of temperature and are not restored upon cooling in synthetic air (Figure 6a). On the postheated ZnO sample, new and redshifted OH bending modes suggest OH species with weaker cation interaction. We attribute the spectral changes seen in Figure 6 to a decreased fraction of lattice defects on the as-prepared sample (Figure 7a), whereby heating the sample leads to irreversible loss of OH and hydride species bonded to lattice defects (O vacancies and Zn interstitials), while on the postheated sample (Figure 7b), containing fewer lattice defects, reversible dissociative adsorption of O_2_ and H_2_O depletes and replenishes OH groups on the ZnO nanorods and dominates the spectral features. The schematic illustration in Figure 9 depicts possible surface OH groups that are hypnotized to form near surface defects on ZnO, both in a simplified one-dimensional view (upper panel) and in a 3D view of a hexagonal surface (lower panel), exposing oxygen and Zn vacancies, respectively.

**Figure 9:**
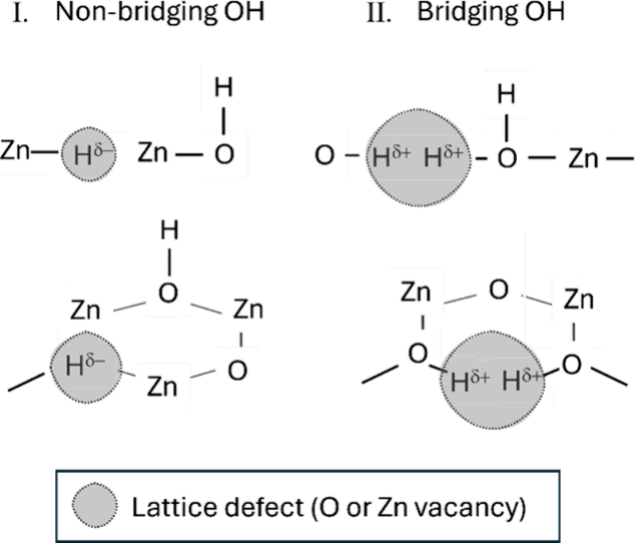
Schematic illustrations of different types of surface −OH groups bonded near defect sites on the ZnO surfaces: side view (top) and hexagonal polar (bottom); (I) O vacancy site and (II) Zn vacancy site.

Supporting these conclusions, Figure 10a shows enlarged DRIFT spectra in the 3000–4000 cm^–1^ regions (showing the O–H stretching bands) of the as-prepared ZnO sample acquired at 450 °C. The sample was subjected to a 25 °C/min heating ramp in synthetic air from 20 to 450 °C, and spectra were collected in situ during the heating ramp. Loss of absorbance indicates disappearance of a vibrational mode associated with hydroxyls in a different chemical environment. Typical peaks found on ZnO nanoparticles are observed.^32,46^ The strong negative band bands at 3468 and 3572 cm^–1^ are associated with hydroxyl groups on defect sites.^32^ Simultaneously, as the ZnO sample is heated and water is desorbed from the catalyst, bands at about 3646 and 3667 cm^–1^ start to be distinguished, which can be attributed to isolated hydroxyls.

**Figure 10:**
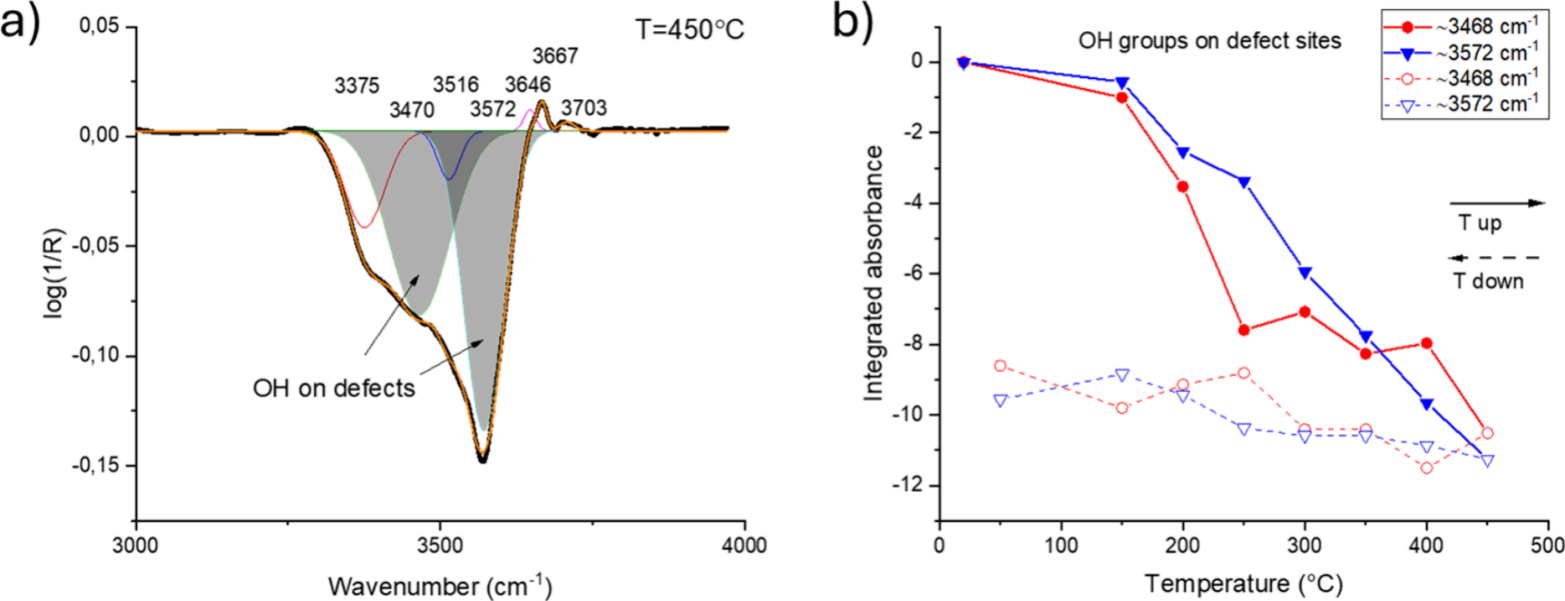
(a) In situ DRIFT spectrum between 3000 and 4000 cm^–1^ of as-prepared ZnO after heating in situ to 450 °C applying a temperature ramp of 25 °C/min in synthetic air starting from room temperature. (b) Integrated absorbance of OH bands associated with defects as a function of sample temperature acquired by operando DRIFTS. Spectra between 4000 and 3000 cm^–1^ were deconvoluted using supervised Gaussian peak fitting yielding a coefficient of determination *R* ^2^ > 0.999 with <4 cm^–1^ peak maximum variation throughout the whole temperature series.

Figure 10b shows the integrated absorbance of peak deconvoluted OH bands at 3468 and 3572 cm^–1^ as a function of sample temperature as determined by operando DRIFTS. It is evident that the absorbance of defect-associated OH bands decreases irreversibly by heat treatment and is not restored by cooling down in air. We attribute this to reduction of surface defects as a function of temperature, in agreement with the analysis of the fingerprint region in Figure 6 showing irreversible loss of bridging OH species, as well as TEM and XRD data showing progressively more well-developed facets as a function of heat treatment.

### Photocatalytic Decomposition of Phenol

The photocatalytic activities of the four ZnO samples, as-prepared and heat-treated at 500 °C for 10, 30, and 60 min, were evaluated by following the decomposition of phenol under UV illumination (λ = 365 nm). The UV illumination started after 30 min of phenol addition allowing steady-state conditions to be established. No significant differences in adsorption capacity among samples were observed. Phenol was used as a model pollutant for several reasons: (1) it does not act as a visible-light sensitizer, (2) phenol is easily detected by UV spectrometry, and (3) a decrease in the UV absorption band can unambiguously be attributed to degradation of the aromatic ring. Typically, the degradation follows first-order reaction kinetics when spectrophotometrically analyzing the disappearance of the OH-modified π–π* transition in the phenol molecule around 270 nm.^47,48^

Figure 11a shows the phenol concentration as a function of UV illumination time for the ZnO samples. The photodegradation experiment started after an initial 30 min equilibration time in the phenol solution (data not shown). It is evident that the as-prepared and the 10 min heat-treated ZnO have the lowest reaction rates. The as-prepared ZnO sample exhibits an about 45 min “induction period” where no measurable phenol degradation occurred. We attribute this induction period to a period where surface-bonded organics and carbonates are decomposed, eventually exposing active sites available for reaction with phenol. The other three catalysts, heat-treated for different times at 500 °C, displayed an immediate decomposition of phenol. The disappearance of phenol concentration is well-described by first-order reaction kinetics as seen in Figure 11b, similar to previous studies.^47,48^ We measured the concentrations of phenol, catechol, and hydroquinone by HPLC for the as-prepared ZnO and the ZnO sample heated at 500 °C for 30 min (see the Supporting Information). Neither catechol nor hydroquinone was detected at the beginning of UV illumination but started to appear first after 15 min and increased with time (Figure S2), thus supporting the well-known phenol photooxidation pathway initiated by OH^·^ radicals,^49^ viz., phenol → catechol → hydroquinone → ring opening → ... → RCOO → CO_2_ + H_2_O.

**Figure 11:**
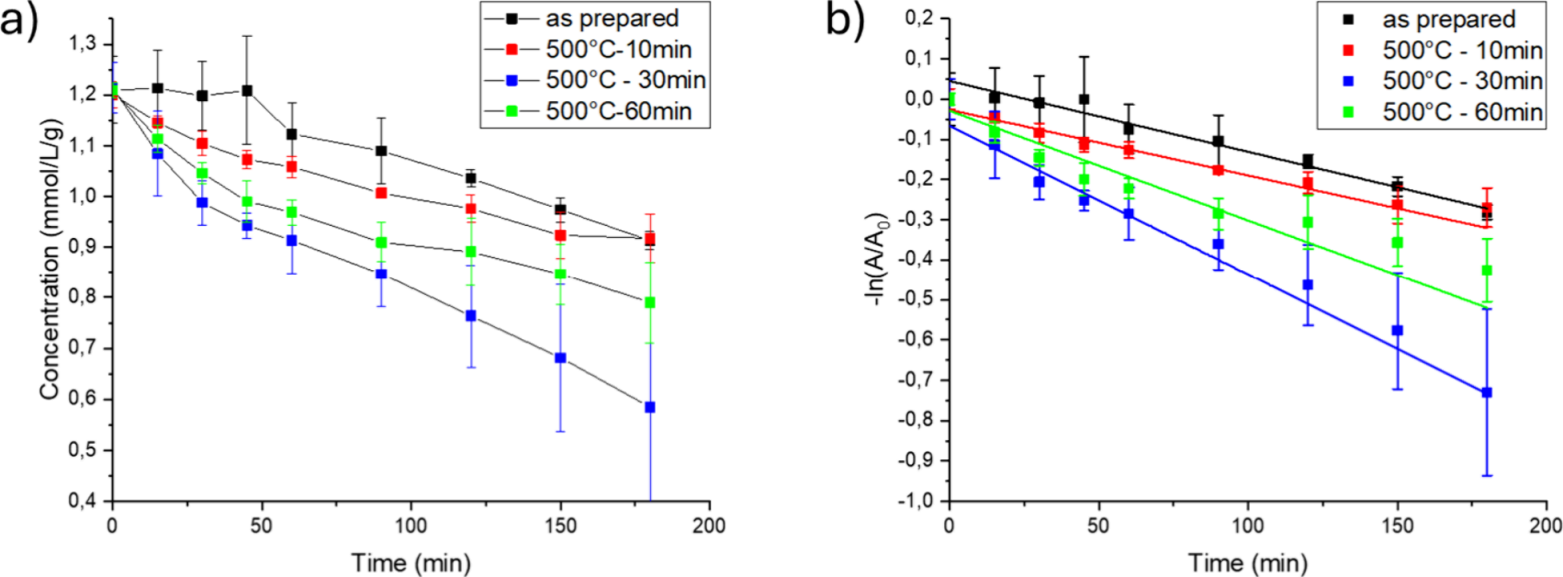
(a) Photocatalytic degradation of phenol per gram of the ZnO catalyst as a function of UV illumination for as-prepared ZnO and ZnO postheated at 500 °C for 10, 30, and 60 min. The reported data are average values from three replicates. Error bars represent standard deviation. (b) Corresponding pseudo-first-order reaction kinetic plots ln­(*A*_0_/*A*) vs time.

There are several studies on the photocatalytic decomposition of phenol on ZnO nanoparticles. However, these studies are in general not directly comparable due to differences in experimental conditions. The optimum amount of a catalyst or illumination conditions are usually not established, or optimized, and the reported results vary considerably.^50^ Further, the pH of solution affects the measured rate. Neutral to slightly acidic pH has been reported to be beneficial. The same is true for this work, where our focus is on surface properties, not on chemical reaction engineering. Nevertheless, to put our work in perspective, we compare our results summarized in Table 2 with some literature reports that are sufficiently similar to ours. Jiang and co-workers^51^ used 1 g/L ZnO “nanoflowers” to decompose 0.27 mM phenol under simulated solar illumination and observed about 40% degradation after 2 h. Uribe-Lopez et al.^52^ used 1 g/L rod-like ZnO, obtained by means of sol–gel or precipitation to decompose a solution of 0.53 mM phenol under UVA illumination. They observed 100% degradation for the precipitated ZnO and 80% degradation for the sol–gel synthesized ZnO after 2 h.

**Table 2:** Photocatalytic Degradation Rates of Phenol for ZnO Nanoparticles Heat-Treated at Different Times at 500 °C

ZnO sample	rate constant μmol min^–1^ m^–2^ (μmol min^–1^ g^–1^)	phenol photodegradation after 180 min of UV illumination
as-prepared	0.26 (1.9)^a^	24%
annealed for 10 min	0.23 (1.5)	23%
annealed for 30 min	0.59 (3.6)	52%
annealed for 60 min	0.24 (2.1)	34%

a Not including the initial 45 min dwell time that shows no phenol degradation.

While heat treatment at 500 °C yields improved crystallinity according to XRD and TEM data, we infer that this effect cannot be the main reason for the results shown in Figure 11. For example, at an intermediate heat treatment time of 30 min, a significant increase in the degradation rate and total amount of degraded phenol (>50%) is observed. The surface area normalized degradation rate shows that the ZnO sample heat treatment at 500 °C for 30 min exhibits about twice the reaction rate compared with the other samples (Table 2). In contrast, the differences in crystallinity and surface area as a function of heat treatment time at 500 °C seen in Table 1 do not correlate with the data in Figure 11. However, heat treatment removes synthesis residues originating from the acetate precursor. Notably, the surface concentration of carboxylates significantly reduces after heat treatment in air (Table 1). Removal of these species liberates adsorption sites on ZnO and is expected to enhance the catalytic activity of the catalyst. This argument explains qualitatively the increase in the reaction rate versus heat treatment, but it cannot explain the declined reaction rate for the ZnO sample subjected to the longest heat treatment time (60 min at 500 °C). Instead, we infer from the results in Figures 7, 9, and 10 that hydroxyls associated with defects decreases upon heat treatment. Defects play a major role in the photocatalytic activity of ZnO, where bulk defects are reported to have a detrimental effect, acting as recombination centers, while surface defects generally enhance the catalytic effect.^53–56^ In photocatalysis, hydroxyls can assist to separate electron–hole pairs by trapping holes. Studies have shown that along with the surface area and morphology, the amount of surface defects is important in determining the catalytic activity.^53^ Surface defects can be created by the removal of surface ligands, e.g., via thermal removal during heating of the catalyst. On the other hand, heat treatment in air can also decrease the number of oxygen vacancies, leading to lowered photocatalytic activity with increased temperature.^57^ The data shown in Figures 7 and 10 show that OH groups bonded to defects are irreversibly removed by heat treatment of ZnO. Together with the data shown in Figure 11, we can see that extended heat treatment deteriorates the degradation rate. From this reasoning, we conclude that the ZnO annealed at 500 °C for 30 min represents a “sweet spot” where much of the surface organics have been removed while still leaving active surface defects (OH groups bonded to defects) that promote the catalytic activity. On the other hand, the 10 min sample exhibits predominant poisoning by synthesis residues, while the 60 min sample has lost much of the surface defects during extended heating, thus reducing the catalytic activity.

## Conclusions

ZnO nanorod assemblies were synthesized by a commonly employed wet chemical method to investigate their surface properties as a function of pretreatment conditions. It was found that as-prepared ZnO prepared from the acetate precursor contains large amounts of organic residues, even after extensive washing with water, that deteriorate the catalytic activity. Heating at 500 °C was efficient in removing these organic species and simultaneously improving the crystalline structure and facet morphology. However, prolonged heat treatment in air resulted in a decrease in photocatalytic activity, despite maintaining approximately the same particle size and morphology. This is due to irreversible loss of OH groups bonded to surface defects, which are active sites in the phenol photooxidation. It is concluded that there is a balance between removal of site-blocking organic residues and healing of active surface defect sites. By choosing appropriate sample postannealing, it is possible to remove site-blocking organic residues, while retaining chemically active surface OH groups. Our results shed light on previous reports that empirically have shown that surface treatment and hydroxyl groups contribute significantly to the photocatalytic reactivity of ZnO materials. We conjecture that other pretreatment methods, such as high pH preconditioning combined with reduction treatments, also could be applicable for judiciously removing organic residues while still maintaining a high concentration of surface defects, methodologies that would be valid also using other organometallic precursors for the ZnO synthesis.

## Supplementary Material



## Notes

UV spectra of phenol solutions with ZnO, HPLC analysis of reaction products, and concentration of phenol and main intermediate reaction products measured by HPLC (PDF)
