# Supplementary material for: Effect of Surface Impurities and Lattice Defects on the Photocatalytic Activity of ZnO Nanoparticles
Source: Langmuir. 2025 Oct 16;41(42):28399–410. doi: 10.1021/acs.langmuir.5c03385 (PMC12573794; doi:10.1021/acs.langmuir.5c03385)
Supplement: Supplementary file 1 [file la5c03385_si_001.pdf]

Supporting Information

Effect of surface impurities and lattice defects on the photocatalytic activity of ZnO nanoparticles

Fredric G. Svensson[1], Erik Djurberg[1], Seohan Kim[2], Gunnar Westin[3], and Lars Österlund[1,4]*

[1] Department of Materials Science and Engineering, The Ångström Laboratory, Uppsala University, Uppsala 751 03, Sweden.

[2] Department of Materials Science and Engineering, Pusan National University, Busan 46 241, South Korea.

[3] Department of Chemistry, The Ångström Laboratory, Uppsala University, Uppsala 751 21, Sweden.

Correspondence: lars.osterlund@angstrom.uu.se

[4] Department of Chemistry, Umeå University, Umeå 901 87, Sweden.

UV-Vis spectroscopy of phenol in aqueous ZnO solutions

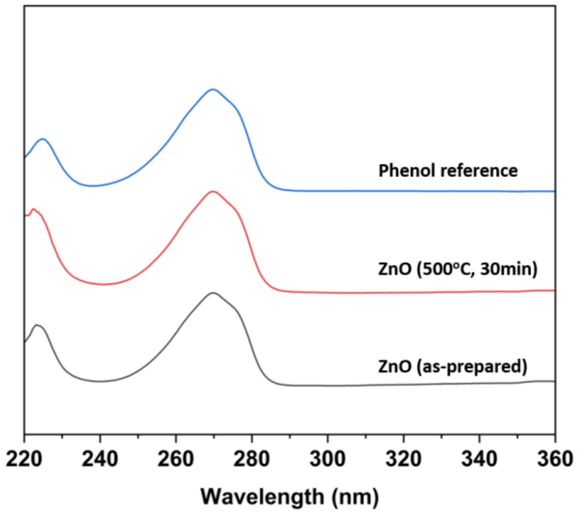

Figure S1. UV-spectra of phenol solutions with ZnO (as-prepared and heated at 500°C for 30 min) at time 0 min and pure reference phenol solution (60 ppm) indicating no interference of ZnO UV-absorption.

HPLC analysis of reaction products

The HPLC analyses for phenol (99%, Aldrich-Sigma) degradations products was made using a Shimadzu system (Shimadzu, Japan) equipped with a photodiode array detector and a 150 mm × 4.6 mm × 2.7µm-Ascentis Express 90 A° AQ-C18 column (Supelco Merck). The mobile phase used was a 95 %v / 5%v acidified water (with 0.1%w/w HCOOH) / methanol mixed solution with a flow-rate of 1.0 mL min^{-1}. The column working temperature was set at 40°C. The phenol and intermediate products were measured before and after regular illumination times up to 180 min of UV illumination (365 nm; 4.8 mW/cm^2) using a pre-heated UV-A PL-L 18W (Philips) light source. The concentration of the photocatalysts (as prepares and post-annealed ZnO) was 1 $g{\cdot}L^{-1}$. An aliquot of 0.8 mL of the solution was sampled and filtrated on an MILLEX HVLP 0.45 µm hydrophilic filter (Millipore, Burlington, MA,

USA) for each HPLC analysis. The results of the calibrated HPLC analysis are shown in Fig. S2 for an intimal concentration of 60 ppm phenol. The results support a reaction pathway, as follows:

phenol → catechol → hydroquinone → ring opening → …→ RCOO→CO_2+H_2O,

which is the expected result for photocatalytic treatments of phenol solutions due to reactions of phenol with OH and O_2 radicals.

a) b)

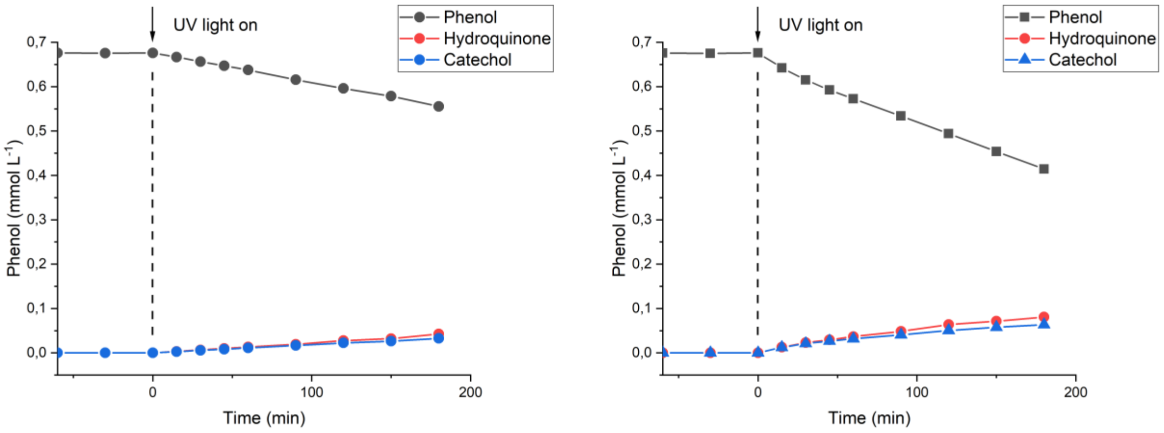

Figure S2 Concentration of phenol and main intermediate reaction products measured by HPLC for a) as-prepared ZnO, and b) ZnO post-heated for 30 min at 500°C.
